# Development and Validation of a Practical Nutritional Management Algorithm in Malabsorption

**DOI:** 10.3390/nu18111750

**Published:** 2026-05-29

**Authors:** Maryam Sidahi Serrano, Carmelo Diéguez Castillo, Andrea Martín Aguilar, Daniel De Luis Román

**Affiliations:** 1 Internal Medicine Department, Infanta Elena Hospital, 21007 Huelva, Spain; sidahi@hotmail.com; 2 Specialist in Gastroenterology, Torrecardenas University Hospital, 04009 Almeria, Spain; 3 Abbott Laboratories, Medical Department, 28050 Madrid, Spain; andrea.martin@abbott.com; 4 Center of Investigation Endocrinology and Nutrition, Valladolid University Clinical Hospital, Medicine School University, 47002 Valladolid, Spain; dadluis@yahoo.es; 5 Health Research Institute of Valladolid (IBioVALL), Valladolid University Clinical Hospital, 47002 Valladolid, Spain; 6 Center for Biomedical Research Network (CIBEROBN) of Obesity and Nutrition, Instituto de Salud Carlos III, 28029 Madrid, Spain

**Keywords:** oligomeric enteral formulas, malabsorption, algorithm, protocol, gastrointestinal symptoms, gastrointestinal tolerance, nutritional management

## Abstract

Background: Malabsorption is a frequent and clinically relevant condition associated with a high risk of disease-related malnutrition across multiple gastrointestinal and systemic disorders. Despite its prevalence, standardized nutritional management algorithms remain limited. Following a previously published Delphi consensus on the use of oligomeric enteral formulas, the present study aimed to develop and validate a practical nutritional management algorithm for patients with malabsorption. Methods: A structured expert questionnaire was conducted among 173 physicians with clinical experience in malabsorption, including specialists in endocrinology, gastroenterology, internal medicine, and oncology. Participants gained experience using the preliminary algorithm by applying it to five real-life cases before completing the questionnaire. The survey addressed symptom type, intensity, and duration required for screening, diagnostic criteria for malnutrition, timing of reassessment, indications for oligomeric oral nutritional supplements (ONSs), and criteria for reintroducing polymeric formulas. Statistical analyses were performed using SAS v9.4. Results: Of the 173 participants, 67.1% were women, with a mean age of 39.6 ± 8.2 years and a mean clinical experience of 10.9 ± 7.9 years. In clinicians’ opinion, diarrhea was the most frequently considered symptom to trigger screening (88.6%), followed by abdominal distension (72.6%), abdominal pain (65.4%), and increased gastric residuals (45.8%). Experts agreed that symptoms should present with at least moderate intensity and persist for more than 3 weeks to justify screening. Most respondents agreed with using the GLIM criteria for malnutrition assessment (97.7%). For patients with poor tolerance to polymeric ONSs or moderate-to-severe symptoms, initiation of oligomeric ONSs was recommended, with diarrhea identified as the main indication (31.1%). Symptom severity prompting oligomeric formulas was rated between 2.8 and 3.3 on a 5-point scale. The mean recommended duration of symptom improvement before transitioning back to polymeric formulas was 6.24 ± 4.45 weeks. Conclusions: This study presents a consensus-based, clinically applicable algorithm for nutritional screening, diagnosis, and intervention in patients with malabsorption. The algorithm provides clear guidance on symptom assessment, use of GLIM criteria, selection of ONS type, and follow-up, potentially improving standardization and quality of nutritional care in this high-risk population.

## 1. Introduction

Malabsorption is an important challenge in the management of patients with gastrointestinal disorders, digestive tract surgery, and serious or critical illnesses, and results in a considerable increase in the risk of malnutrition [1]. Many gastrointestinal diseases are associated with problems related to malabsorption, which can be caused by dietary problems, alterations in nutrient absorption, digestive enzyme insufficiency and other related conditions [2,3]. Such patients may experience diarrhea, abdominal pain or distension, increased gastric residual volume, or vomiting. Malabsorption has a severe impact on the patient and may lead to malnutrition.

The prevalence of malnutrition varies depending on the patient’s profile. In patients with inflammatory bowel disease (IBD), the prevalence ranges from 6.1 to 69.7% [4]. Likewise, malabsorption is a common complication of different types of surgical resections of the gastrointestinal tract, particularly in bariatric surgery. Malabsorption is common after esophagogastric cancer surgery, and affects 78% of all post-gastrectomy patients [5]. In individuals with esophageal and gastric cancer, malabsorption is evident in 73% of cases, and 44% suffer pancreatic insufficiency [6]. Bariatric surgery, especially malabsorptive procedures such as Roux-en-Y gastric bypass operations, can lead to significant nutritional deficiencies [7]. Despite its clinical relevance, there is no clear consensus on the symptoms that define malabsorption, as these can vary considerably depending on the etiology and severity.

We have previously published a consensus study [8] using the Delphi methodology with the primary objective of establishing the role of oligomeric enteral formulas in the nutritional management and nutritional status of patients with malabsorption syndrome. The study involved experts from different specialties with extensive clinical experience, which allowed for identifying areas of agreement in a setting characterized by a lack of high-quality clinical evidence. The consensus reached showed that oligomeric enteral formulas represent a nutritional strategy particularly indicated for patients with malabsorption and moderate-to-severe gastrointestinal symptoms, such as persistent diarrhea, abdominal distension, abdominal pain, or poor tolerance to polymeric formulas. Likewise, their usefulness as an initial treatment to improve gastrointestinal tolerance, promote nutrient absorption, and facilitate the recovery of nutritional status in stages of greater functional impairment of the gastrointestinal tract was also stressed.

The consensus also emphasized the need for structured clinical protocols to guide decision-making in daily practice, including clear criteria for the initial indication of oligomeric formulas, clinical monitoring of the response, and progressive and controlled transition to polymeric enteral formulas once symptom improvement has been achieved. However, although the Delphi study allowed for harmonizing expert opinion and defining theoretical recommendations based on accumulated clinical experience, its methodological nature did not allow direct assessment of the practical applicability or operability of the proposed algorithm in real-life healthcare scenarios. For this reason, the clinical efficacy of the initial algorithm and its usefulness in daily care practice required additional validation in a real-life clinical setting, allowing the adjustment of those aspects with a lower level of evidence, improving operational clarity, and facilitating its implementation by professionals from different specialties. The present study thus aims to present the final version of the nutritional management algorithm for patients with malabsorption, prepared based on practical experience and expert consensus, and aimed at all professionals involved in the treatment of patients with diseases causing malabsorption.

## 2. Materials and Methods

Following the development of a first version of the consensus by the coordinating group, based on the results of the previously published Delphi study [8], a subsequent phase was designed to assess the clinical applicability and operability of the proposed algorithm in a real-world care setting. To this end, a total of 173 experts with experience in the management of patients with malabsorption participated in the practical validation of the initial algorithm (β version), applying it systematically in five real-life patients with suspected or confirmed malabsorption. It is important to emphasize that this study phase did not involve the collection of clinical data or personal information from the patients themselves. The application of the algorithm to five real-life cases was designed exclusively as a practical exercise to provide physicians with direct experience in the tool’s usage and clinical flow. This firsthand experience ensured that their subsequent responses to the structured questionnaire regarding the algorithm’s operability and functionality were based on practical knowledge and clinical judgment rather than theoretical assumptions.

Following this clinical application phase, the participants were given a structured questionnaire to record their experience in using the algorithm and to explore those aspects of nutritional management with less supporting scientific evidence. The questionnaire included specific questions related to the clinical criteria required to start nutritional screening, such as the predominant symptoms, their severity, and the minimum duration required to consider suspected malabsorption as being clinically relevant. Likewise, an evaluation was made of those aspects related to the timing of clinical re-evaluation, the most appropriate time to start treatment with oligomeric enteral formulas, and the clinical criteria to be met in order to consider a safe transition to polymeric enteral formulas.

In addition, the questionnaire addressed practical items intended to improve the applicability of the algorithm, including the most appropriate diagnostic tool for detecting malnutrition, the frequency of clinical and nutritional controls, the symptoms to be prioritized during follow-up, and the optimum time to assess tolerability of the nutritional treatment. In order to ensure adequate understanding of the algorithm and to standardize the response criteria, the participants were asked to complete the application of the algorithm in the proposed five clinical cases before answering the questionnaire.

**Figure 1:**
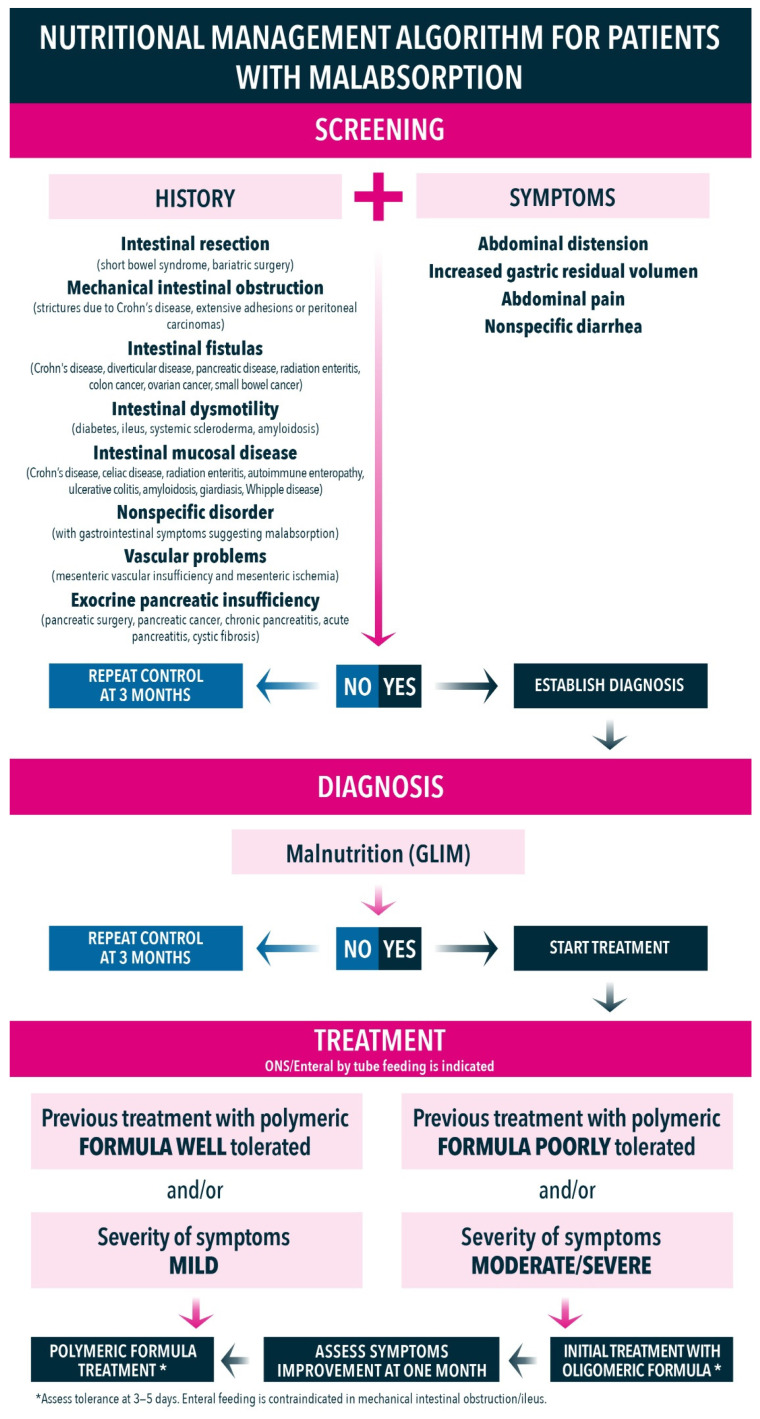
Nutritional management algorithm for patients with malnutrition.

Based on the analysis of the responses obtained, the coordinating group reviewed and adjusted the initial algorithm, incorporating the input from the experts and resolving the points of uncertainty detected during the clinical validation phase. This process led to the preparation of the final version of the algorithm for the nutritional management of patients with malabsorption (α version), which is presented in Figure 1. In summary, after a first theoretical consensus phase (β algorithm) and a second practical validation phase under real-life clinical condition, a final version of the algorithm was developed offering greater operational clarity and clinical applicability. The purpose of this study is to present this final version of the algorithm, aimed at all professionals involved in the nutritional management of patients with diseases causing malabsorption.

### 2.1. Participants

Specialists in endocrinology, gastrointestinal tract disorders, internal medicine, and oncology, all with clinical experience in patients with disease-related malnutrition and malabsorption syndrome, participated in the study. All participants were familiar with the initial algorithm (β version) and conducted their care activity in clinical settings where the nutritional management of malabsorption is part of routine practice (Table 1).

Regarding the selection of the study population, inclusion criteria required participants to be medical specialists with documented clinical experience in the management of disease-related malnutrition and malabsorption syndrome. Exclusion criteria were applied to those physicians who did not routinely manage nutritional support or whose regular clinical practice did not involve the care of patients with malabsorption. All participants were hospital-based specialists (practicing in both inpatient and specialized outpatient settings) from centers where the nutritional management of malabsorption is part of routine care protocols. Many of these individuals are members of multidisciplinary expert teams or hospital nutrition committees. The distribution of medical specialties, shown in Table 1, reflects the clinical organization of the participating centers. Endocrinologists (50.2%) and Gastroenterologists (25.4%) represent the largest groups, as they are the primary specialists responsible for the management and specific nutritional control of malabsorption symptoms in these institutions. Furthermore, the study included specialists from Internal Medicine (8.6%) and Oncology (10.4%), as these departments also frequently manage patients with systemic disorders or neoplastic conditions—such as esophagogastric cancer—that are highly associated with malabsorption and require specialized nutritional intervention.

### 2.2. Statistical Analysis

Statistical analysis was performed using the Statistical Analysis System (SAS) version 9.4 package. Qualitative variables were analyzed using the nonparametric Fisher’s exact test, while quantitative variables were compared using the Kruskal–Wallis test, as normal distribution could not be assumed. Comparative analyses between the different medical specialties were also performed to identify possible differences in the application of the algorithm and in the clinical criteria used. Statistical significance was considered for *p* < 0.05.

## 3. Results

### 3.1. Results of the Expert Questionnaire

A total of 173 physicians participated in the study, 67.1% of whom were women, and the mean age was 39.6 ± 8.2 years. The number of years of experience was 10.9 ± 7.9 (mean ± SD [standard deviation]) (Table 1).

**Figure 2:**
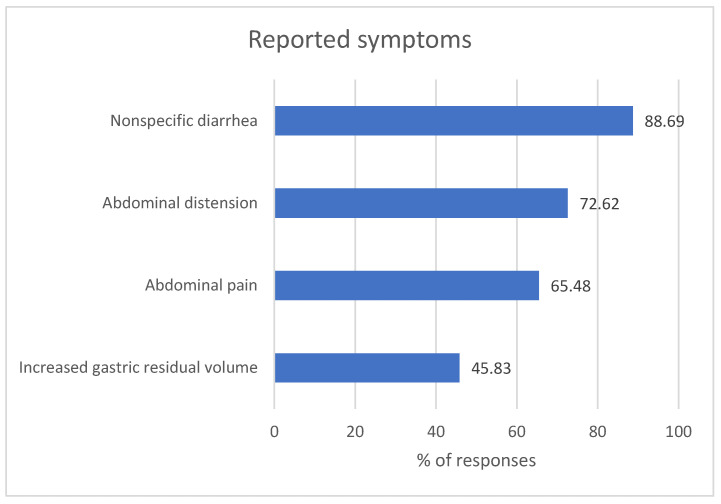
Reported symptoms for starting screening.

**Figure 3:**
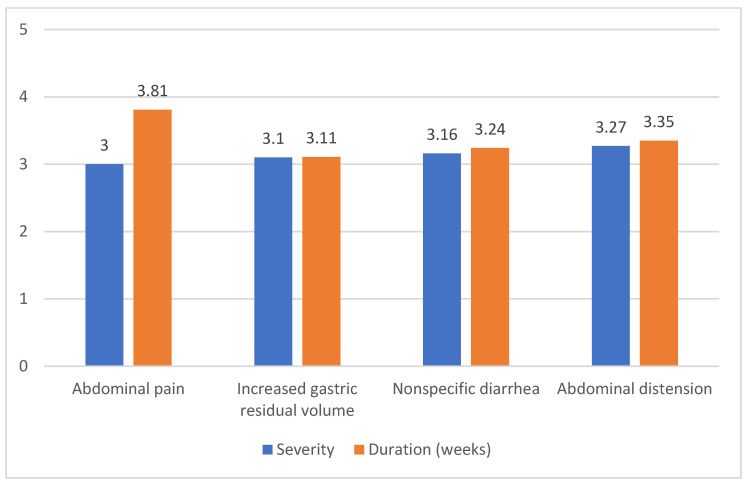
Severity and duration of symptoms for starting screening. Severity 1: Mild, 5: Very severe. Duration in weeks.

With regard to the question related to the number, severity, and duration of symptoms at which they think patients should present to be screened, 88.6% reported diarrhea, 72.6% abdominal distension, 65.4% pain, and 45.8% increased gastric residual volume (Figure 2). In relation to the combination of symptoms, the three most commonly reported were increased gastric residual volume, nonspecific diarrhea, abdominal distension and pain with 28.2% mentions, followed by nonspecific diarrhea, abdominal distension and pain with 25.2%, and finally nonspecific diarrhea with 12.2%. In all the specified symptoms, the specialists indicated that in order to be screened, a patient should at least present medium intensity and a symptom duration lasting for more than 3 weeks (Figure 3).

Most respondents agreed with using the GLIM (Global Leadership Initiative on Malnutrition) criteria for malnutrition assessment (97.7%). With regard to the time when patient risk should be reassessed, if the result proved negative, 68.7% indicated at three months, 30.5% at six months, and 0.8% at one year.

To improve reproducibility and reduce interobserver variability, gastrointestinal symptom severity was operationally categorized according to its clinical impact. Mild symptoms were defined as occasional manifestations with minimal interference in oral intake or daily activities. Moderate symptoms were considered those that persist over time and require dietary adaptations or partially reduce oral intake. Severe symptoms were defined as those associated with marked impairment of oral intake, dehydration risk, significant nutritional deterioration, or the need for medical intervention. These categories are intended to provide practical clinical guidance while preserving flexibility for individual patient assessment.

**Figure 4:**
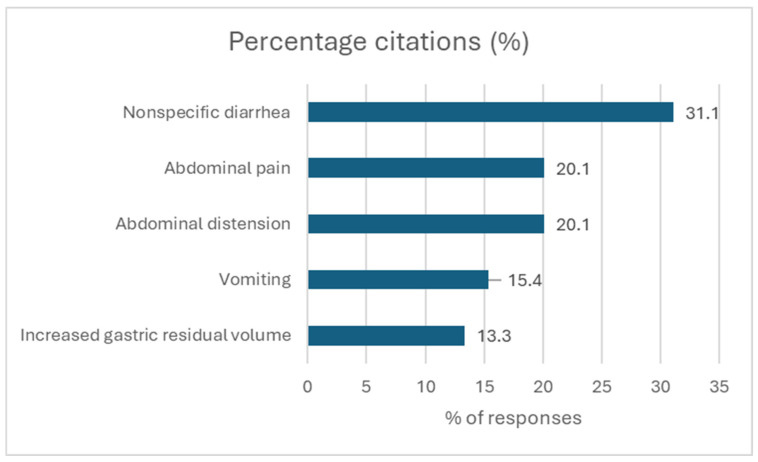
Symptoms that guide the use of oligomeric ONSs.

**Figure 5:**
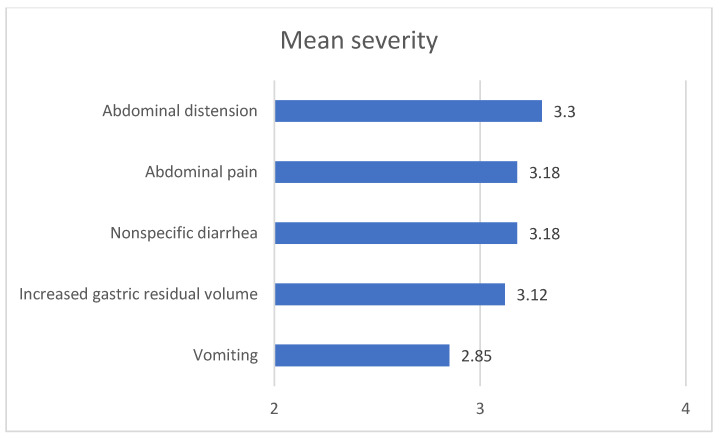
Mean severity of symptoms that guide the use of an oligomeric formula. 1: Mild, 5: Very severe.

**Figure 6:**
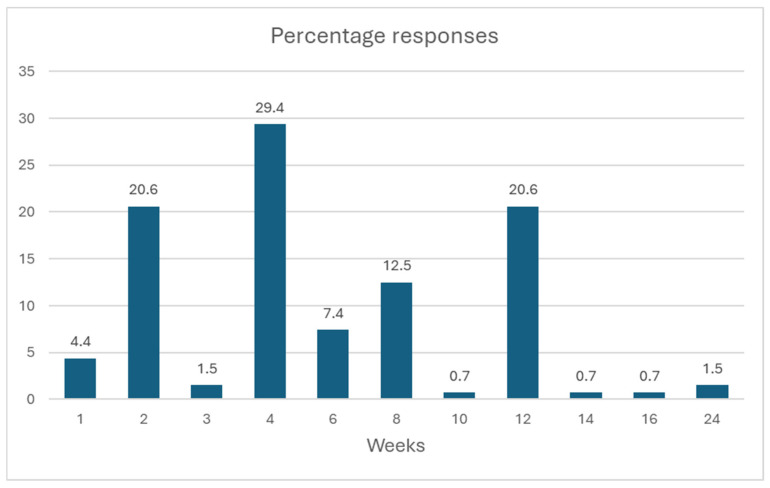
Suggested duration of improvement with ONSs before switching to a polymeric formula.

Regarding the symptoms and severity directly affecting nutritional status and which status should prompt the start of oligomeric oral nutritional supplements (ONSs), 31.1% cited diarrhea, 20.1% abdominal distension, 20.1% pain, 15.4% vomiting, and 13.3% increased gastric residual volume (Figure 4). Nonspecific diarrhea was the symptom most commonly reported by the specialists in endocrinological and in gastrointestinal disorders, while abdominal pain was the symptom highlighted by oncologists. These differences were statistically significant. With regard to the severity of these symptoms for starting an oligomeric formula, on a scale of 1 to 5 points, where 1 is mild and 5 is very severe, abdominal distension scored 3.3 points, diarrhea with 3.1, abdominal pain with 3.1, increased gastric residual volume with 3.1, and vomiting with 2.8 points (Figure 5). In relation to how long symptom improvement should persist with oligomeric ONSs in order to switch to a polymeric formula, the reported mean was 6.24 ± 4.45 weeks (Figure 6). The specialists in gastrointestinal disorders on average required a longer duration to make this change, though the difference was not statistically significant.

### 3.2. Nutritional Algorithm in Malabsorption

After examining the results of the survey, the coordinating group adjusted the initial protocol (β) to the final version (α). The algorithm consists of three stages: screening, diagnosis, and nutritional intervention.

Screening would be required of those patients with a history of intestinal resection (short bowel syndrome, bariatric surgery), mechanical intestinal obstruction (Crohn’s disease, extensive adhesions, peritoneal carcinomas), intestinal fistulas (Crohn’s disease, diverticular disease, pancreatic disease, radiation enteritis, colon cancer, ovarian cancer, small bowel cancer), intestinal dysmotility (diabetes, ileus, systemic scleroderma, amyloidosis), intestinal mucosal disease (Crohn’s disease, celiac disease, radiation enteritis, autoimmune enteropathy, ulcerative colitis, amyloidosis, giardiasis, Whipple disease), vascular problems (mesenteric vascular failure, mesenteric ischemia), nonspecific disorders with gastrointestinal symptoms, or exocrine pancreatic insufficiency (pancreatic surgery, pancreatic cancer, acute or chronic pancreatitis, cystic fibrosis). These patients should be screened when they experience symptoms of abdominal distension, increased gastric residual volume, abdominal pain or nonspecific diarrhea, and if screening proves negative, it should be repeated after three months.

If screening proves positive, the next step is the diagnosis of malnutrition based on the GLIM criteria [9]. The GLIM criteria for the diagnosis of malnutrition provide a framework based on three phenotypic items (unintentional weight loss, low body mass index, and decreased muscle mass) and two etiological items (decreased food intake/assimilation, and inflammation/disease burden). To be diagnosed with malnutrition, a person must meet at least one phenotypic criterion and one etiological criterion. If the GLIM criteria prove positive, treatment should be started and, if negative, reassessment should be performed after three months.

If the patient has been previously treated with polymeric ONSs with good tolerance and/or the symptoms are mild, polymeric ONSs should be continued. If the patient has been treated with polymeric ONSs with poor tolerance and/or the symptoms are moderate/severe, initial treatment with oligomeric ONSs is indicated, and symptom improvement should be assessed after one month. In the event of a favorable course, the patient can be switched to polymeric ONSs. Tolerance should be assessed at 3–5 days after the start of both polymeric ONSs and oligomeric ONSs. The transition from oligomeric to polymeric formulas should not be based exclusively on chronological criteria. Beyond the approximate timeframe identified by expert consensus, transition should be considered only after sustained clinical improvement, including reduction in gastrointestinal symptoms, stabilization or recovery of oral intake, absence of clinically significant vomiting or high-output diarrhea, and adequate tolerance of nutritional support during consecutive follow-up assessments. The proposed timeframe therefore represents practical guidance rather than a rigid mandatory interval.

## 4. Discussion

There is a consensus that the presence of malnutrition should be routinely assessed in all patients with malabsorption [9], since these individuals are at very high risk of suffering from this condition [10]. However, the absence of protocols limits the application of this strategy to some degree [9].

The proposed algorithm emphasizes the systematic assessment of nutritional status which is fundamentally linked to the pathophysiological state of malabsorption. While the association between malabsorption and malnutrition risk is established, the developed protocol interprets this risk as a multifactorial phenomenon. This risk is not only determined by the nature of the underlying disease (e.g., inflammatory bowel disease, oncology, or surgical resections), but is also significantly influenced by the nature and severity of symptoms, such as persistent diarrhea or abdominal pain, which often lead to voluntary or therapeutic dietary restrictions. By utilizing the GLIM criteria for diagnosis, the algorithm specifically accounts for these etiological factors—such as reduced assimilation and disease-related inflammation—ensuring that the nutritional intervention addresses the complex interplay between the primary pathology, its treatment, and the patient’s functional status.

The present study has developed an algorithm for the nutritional management of patients with malabsorption and describes how it was created and what it consists of. A questionnaire developed for a group of experts was used to ask about the different parts of the algorithm, seeking to establish a qualified consensus-based opinion—limited high-quality evidence is available for several of these aspects. The final version of the algorithm has been described based on the results of this survey.

In summary, the most important aspects addressed by the questionnaire were the reported symptoms for starting patient screening, their severity and duration, with the symptoms and their mean severity guiding the adoption of an oligomeric formula, and the suggested duration of improvement for reintroducing the polymeric formula.

Regarding the coexistence of gastrointestinal symptoms, although our data identified common combinations, the coordinating group and the expert panel decided not to require a specific association or a cumulative number of symptoms to trigger the screening process. The rationale behind this decision was to prioritize early detection and clinical utility. The algorithm established that the presence of any single symptom (diarrhea, distension, pain, vomiting, or increased residuals), provided it meets the thresholds of moderate intensity and a duration of more than three weeks, is a sufficient clinical indicator to initiate nutritional screening. By focusing on the presence of symptoms as independent triggers rather than requiring a complex coexistence, the algorithm ensures that patients at high risk of malnutrition are identified promptly, avoiding diagnostic delays that could arise from waiting for a more complex symptomatic profile to develop.

Screening for malnutrition usually involves the use of the MUST (Malnutrition Universal Screening Tool) and MNA-SF (Mini Nutritional Assessment–Short Form) tools, with variations depending on the specialty [11,12], while the GLIM criteria are used for establishing the diagnosis [9].

Because of the high risk of malnutrition, these patients should be treated with dietary changes, nutritional supplementation, enteral nutrition, or parenteral nutrition. Oligomeric formulas are a form of nutritional supplementation whose ultimate aim is to facilitate the digestion and absorption of nutrients, improving patient tolerance to nutritional treatment [13]. Currently, the use of peptide-based formulas is an approach for improving tolerance to enteral nutrition. These are enteral formulas containing proteins enzymatically hydrolyzed to dipeptides and tripeptides. These hydrolyzed proteins are typically combined with a higher content of medium-chain triglycerides to produce an enteral formula that is essentially easier to absorb and use [14]. It should be noted that limited evidence has been found in the literature regarding oligomeric formulas in patients with gastrointestinal problems [1,15], and for this reason we consider the adopted methodology (i.e., asking 173 experts for their opinion) to be adequate.

Oligomeric formulas are specifically designed to minimize digestive requirements and maximize absorption in the presence of a compromised gastrointestinal tract. Beyond their protein content—comprising enzymatically hydrolyzed proteins in the form of dipeptides and tripeptides—and their lipid profile, which typically incorporates a high proportion of medium-chain triglycerides (MCTs) to facilitate absorption without the need for complex micellar solubilization, their composition is precisely balanced in other key components. The carbohydrate source generally consists of maltodextrins, which provide an efficient energy source that is easily hydrolyzed and absorbed with a lower osmotic impact than simple sugars. These formulas are also fortified with a complete profile of vitamins and minerals, often provided in highly bioavailable forms to counteract the micronutrient deficiencies frequently observed in malabsorptive states. Regarding their physical properties, oligomeric enteral nutrition is typically formulated to maintain a controlled osmolarity to ensure optimal gastric emptying and prevent osmotic diarrhea, thereby enhancing overall gastrointestinal tolerance [1].

The published studies on oligomeric formulas include an ESPEN Clinical Guideline [1] which reports that formulas containing peptides and medium-chain triglycerides may facilitate absorption in cases of malabsorption or short bowel syndrome. Some studies have also shown the usefulness of peptide formulas in patients with diarrhea, reducing its volume; likewise, in critically ill patients, such formulas could reduce the number of days with adverse effects or gastrointestinal adverse effects [16]. In severe acute pancreatitis, short bowel syndrome and acute liver failure, both polymeric and peptide diets may be used (level of evidence: moderate; grade of recommendation: moderate) [17]. There is limited high-quality evidence on the composition of enteral formulas in patients with chronic pancreatitis. However, there are data suggesting that semi-elemental enteral formulas with medium-chain triglycerides are more suited for jejunal nutrition compared to polymeric formulas. In two of the aforementioned studies, semi-elemental formulas were used with good gastrointestinal tolerance.

Primo et al. showed that the use of a peptide supplement with short-chain triglycerides after having administered total parenteral nutrition exerts a beneficial effect on the biochemical and anthropometric parameters, and on nutritional status, with high compliance and good tolerance [18]. In turn, López-Medina, in malnourished patients with gastrointestinal symptoms, reported high compliance with a hypercaloric, high-protein peptide-based nutritional supplement, resulting in a reduction in the gastrointestinal symptoms and improvement of the patient’s nutritional status [19].

Regarding the assessment of oral and digestive tolerance, the majority of experts agreed that this evaluation should be conducted early, specifically within 3–5 days of initiation. This rapid assessment is essential to maximize treatment adherence and allow for prompt therapeutic adjustments if necessary. It is important to note that no specific standardized questionnaires were designed for this purpose; instead, monitoring the patient’s clinical response is left to the discretion and clinical judgment of each professional.

The main strengths of this study include its innovative nature, based on the systematic integration of the clinical experience of a large panel of 173 specialists with training and routine practice in clinical nutrition and in the management of patients with malabsorption. This approach allowed us to address areas where the available scientific evidence is limited, providing a pragmatic and multidisciplinary view reflecting the reality of patient care. The resulting algorithm is also characterized by its clear, sequential, and easily applicable structure, which facilitates its incorporation into daily clinical practice by professionals from different specialties and care levels, potentially contributing to the standardization of nutritional management in patients with malabsorption.

However, some limitations should be noted. The algorithm has been evaluated in a test and initial validation phase based on real clinical cases, but has not yet undergone prospective, multicenter validation to confirm its clinical impact, reproducibility and effect on hard clinical outcomes. We acknowledge that the current study represents a foundational phase in the validation of the nutritional algorithm. While the protocol was systematically applied by a large and diverse panel of 173 specialists from various hospital settings and specialties (Endocrinology, Gastroenterology, Oncology, and Internal Medicine), the lack of prospective or external validation is recognized as a limitation. This initial validation phase was primarily designed to ensure the operability and clarity of the tool in a real-world care setting before its broader implementation. Therefore, although the high degree of consensus and the successful practical application by experts suggest a solid framework for reproducibility, we agree that future prospective, multicenter studies are essential. Such research will be necessary to evaluate the algorithm’s real-world effectiveness and its direct impact on hard clinical outcomes across diverse patient populations and different healthcare systems.

While the multidisciplinary nature of nutritional support—involving nurses and dietitians—is essential for the comprehensive care of patients with malnutrition, this study focused specifically on medical specialists (physicians). This decision reflects the current regulatory framework in Spain, where the legal authority for clinical prescription resides exclusively with medical specialists. Consequently, these professionals are primarily responsible for the medical decision-making process regarding the selection and transition between different types of enteral formulas. The participation of endocrinologists, gastroenterologists, oncologists, and internists was prioritized to validate the clinical indicators and diagnostic criteria (such as the GLIM criteria) that trigger a change in medical treatment. However, the final algorithm is intended to be a tool for all healthcare professionals involved in nutritional care, and we acknowledge that future validation phases should incorporate the perspectives of nursing and dietetics to further enhance its multidisciplinary applicability.

The proposed algorithm should be interpreted as complementary to existing international nutritional recommendations rather than as an alternative to them. The framework incorporates the GLIM criteria as the standardized basis for malnutrition diagnosis and remains aligned with ESPEN principles regarding nutritional assessment and enteral support. However, unlike current guidelines, which primarily provide general recommendations, the present algorithm offers a sequential and symptom-oriented practical approach specifically focused on patients with malabsorption, including operational guidance for symptom-triggered screening, selection of oligomeric versus polymeric formulas, and structured clinical reassessment. This integration of standardized diagnostic criteria with pragmatic nutritional decision-making represents the principal added value of the proposed framework.

In addition, the expert consensus-based design may be subject to opinion-inherent biases, though these were mitigated by using a structured methodology. Thus, future studies should systematically assess the efficacy of the algorithm in different care settings and patient populations in order to consolidate its usefulness and clinical generalization.

## 5. Conclusions

In conclusion, this study offers a structured and consensus-based algorithm for nutritional screening, diagnosis and intervention in patients with malabsorption, systematically integrating the clinical assessment of symptoms, use of standardized diagnostic criteria, and selection of the type of oral nutritional supplement based on patient tolerance and clinical severity. This approach allows the standardization of care practice and facilitates decision-making in a clinical setting characterized by high complexity and variability. The proposed algorithm may help improve the early detection of malnutrition, optimize nutritional management, and potentially improve the clinical outcomes and quality of life of patients with malabsorption. However, these findings should be interpreted as a first step towards the standardization of nutritional management, since future research should focus on the prospective, multicenter validation of the algorithm in different care settings and patient populations. It would also be of interest to assess its impact on relevant clinical variables, such as the evolution of patient nutritional status, gastrointestinal tolerance, adherence to nutritional treatment, use of healthcare resources, and medium- and long-term clinical outcomes. Other future lines of research include comparison of the algorithm against conventional management strategies, adaptation of the algorithm to specific patient subgroups according to the etiology of malabsorption, and cost-effectiveness analyses of its implementation. Overall, the integration of this algorithm into clinical practice, together with its evaluation in longitudinal and interventional studies, could represent a significant advance towards a more precise, efficient and personalized approach to malabsorption and associated malnutrition.

The proposed algorithm provides a structured and consensus-based framework for nutritional screening and management of patients with malabsorption and may contribute to greater standardization of clinical practice and decision-making. However, its potential impact on nutritional outcomes, gastrointestinal tolerance, healthcare utilization, and patient quality of life should be considered exploratory until confirmed by prospective multicenter interventional studies specifically designed to evaluate clinical efficacy.

## Figures and Tables

**Table 1:** Characteristics of the participants in the survey. SD: standard deviation.

Variable		N	%
Sex	Male	57	(32.9)
	Female	116	(67.1)
Medical specialty	Endocrinology	87	(50.2)
	Internal Medicine	15	(8.6)
	Oncology	18	(10.4)
	Gastrointestinal system	44	(25.4)
	Other	9	(5.1)
Variable		Mean (SD)	
Experience ^1^	Years	10.9 (7.9)	

^1^ Years of experience in the practice of their specialty (after the period of residency or specialty training).

## Data Availability

The data presented in this study are available upon request from the corresponding author. The data are not publicly available due to ethical reasons.
